# Suppression of Nonsense Mutations by New Emerging Technologies

**DOI:** 10.3390/ijms21124394

**Published:** 2020-06-20

**Authors:** Pedro Morais, Hironori Adachi, Yi-Tao Yu

**Affiliations:** 1 ProQR Therapeutics, Zernikedreef 9, 2333 CK Leiden, The Netherlands; PMorais@proqr.com; 2 Department of Biochemistry and Biophysics, Center for RNA Biology, University of Rochester Medical Center, 601 Elmwood Avenue, Rochester, NY 14642, USA; Hironori_Adachi@URMC.Rochester.edu

**Keywords:** stop codon read-through, NMD, small molecules, antisense oligo, suppressor tRNAs, ADAR-catalyzed editing, box H/ACA RNA-guided modification, CRISPR technology

## Abstract

Nonsense mutations often result from single nucleotide substitutions that change a sense codon (coding for an amino acid) to a nonsense or premature termination codon (PTC) within the coding region of a gene. The impact of nonsense mutations is two-fold: (1) the PTC-containing mRNA is degraded by a surveillance pathway called nonsense-mediated mRNA decay (NMD) and (2) protein translation stops prematurely at the PTC codon, and thus no functional full-length protein is produced. As such, nonsense mutations result in a large number of human diseases. Nonsense suppression is a strategy that aims to correct the defects of hundreds of genetic disorders and reverse disease phenotypes and conditions. While most clinical trials have been performed with small molecules, there is an increasing need for sequence-specific repair approaches that are safer and adaptable to personalized medicine. Here, we discuss recent advances in both conventional strategies as well as new technologies. Several of these will soon be tested in clinical trials as nonsense therapies, even if they still have some limitations and challenges to overcome.

## 1. Introduction

The current genetic code consists of a total of 64 different codons (triplets of nucleotides), of which 61 are sense codons coding for 20 amino acids, and the remaining 3 (UAA, UAG. or UGA) are nonsense (or stop) codons that signal the termination of protein synthesis [1]. This code is considered to be nearly universal in translating mRNAs into proteins in vitro and in living organisms.

**Figure 1:**
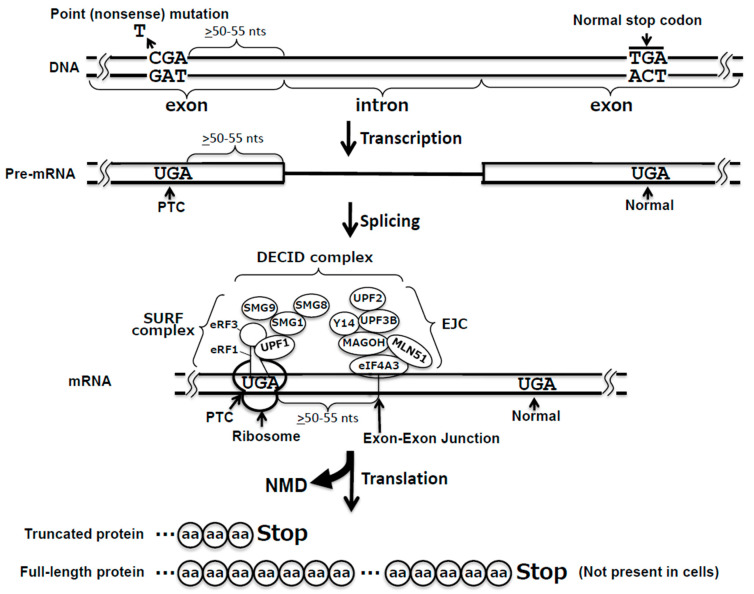
Nonsense mutation and its consequences. A point mutation (indicated) occurs in a gene, creating a premature termination codon (PTC) at the site >50–55 nts upstream of the exon–intron junction (indicated). After transcription, the nonsense mutation is passed onto pre-mRNA. Upon splicing, an exon–exon junction complex (EJC) forms (indicated). When the ribosome encounters the PTC, the eRF1–eRF3 complex enters the ribosome and recruits a number of factors to form the SMG1/UPF1/eRF1/eRF3 (SURF) complex. Together, EJC–SURF combination is called the decay-inducing (DECID) complex. Consequently, nonsense-mediated mRNA decay (NMD) is activated, and the mRNA is degraded. A small fraction of mRNA that escapes degradation is translated, but translation terminates at the PTC, generating a truncated protein (indicated). The normal stop codon is also indicated, and if the PTC is absent, translation will terminate at the normal stop codon, generating a full-length protein (also shown). DNA, pre-mRNA, mRNA, the exons and intron, and the amino acid protein chains are indicated.

Nonsense mutations are a type of point mutations that result in the conversion of a sense codon in the coding region into a nonsense codon, which is also referred to as premature termination codon (PTC) [2]. The effect of nonsense mutations is deleterious on the expression of the affected genes and occurs at two different levels: mRNA decay and premature translation termination (Figure 1). It has been well established that when a translating ribosome encounters a PTC that is at least 50 nucleotides upstream of an exon–exon junction, a surveillance pathway referred to as nonsense-mediated mRNA decay (NMD) is activated, degrading the PTC-containing mRNA [3]. A small fraction of the mRNA transcript that escapes degradation is translated into protein, but translation terminates prematurely at the PTC, resulting in the production of a truncated protein, which is often either non-functional or even harmful to the cell [4]. 

There are a large number of human diseases that result from nonsense mutations in the respective disease genes. The list of such diseases is very long [5]. For instance, Usher syndrome is an inherited retinal dystrophy (IRD) that is the principal cause of combined deafness and blindness. Nonsense mutations occur in 12% of Usher syndrome patients and have been described in different genes, such as the *USH2A* gene [6,7]. Some Hurler Syndrome patients, suffering from skeletal abnormalities and cognitive impairment, carry a nonsense mutation in the *IDUA* gene that prevents the production of a functional full-length IDUA protein in these patients [8]. A substantial fraction of cystic fibrosis (CF) cases, a chronic disease affecting the lungs and the digestive system, is due to nonsense mutations in the *CFTR* gene. The PTCs resulting from these nonsense mutations are identified in the coding region at several different sites, each of which leads to total lack of functional full-length CFTR protein [9]. Nonsense mutations are also found in some relevant oncogenes of many cancer patients, resulting in complete lack of full-length protein products [10]. Given the deleterious role of nonsense mutations in gene expression and disease, nonsense suppression becomes an attractive strategy and the ultimate goal in combating these diseases. 

**Figure 2:**
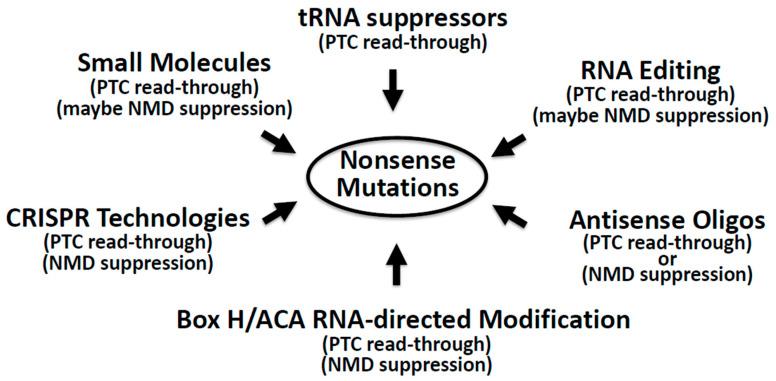
Nonsense suppression by various approaches. A number of approaches (indicated) are developed to suppress nonsense mutations. Some can suppress either NMD or translation termination, while others can suppress both (indicated).

There are several different approaches that have been developed to suppress nonsense mutations. For instance, aminoglycoside antibiotics such as Gentamicin have been used for PTC read-through [11]. Similarly, a small compound named Ataluren has also been tested to suppress nonsense mutations [7,12,13]. Both Gentamicin and Ataluren target the ribosome to induce the read-through of PTC and are fairly well developed. Notably, however, new excitement has risen about developing potential therapeutics (primarily nucleic acids-based approaches) for human diseases resulting from nonsense mutations. These new approaches include (but are not limited to): (1) antisense oligonucleotides to alter the processing of pre-mRNA and to modulate the expression of essential factors for NMD and translation termination, (2) suppressor-tRNAs to read PTC, (3) RNA editing to change A to I at the PTC, (4) box H/ACA guide RNAs to direct modification at the PTC thus converting it back to a sense codon, and (5) the CRISPR technology (Figure 2). All these approaches have their own advantages and limitations, which will be discussed below.

## 2. Translation Termination: Mechanism of Action

The termination codons, UAA, UAG, and UGA, do not have any cognate tRNAs but instead are recognized by release factors. In eukaryotic cells, a single release factor, eRF1, recognizes all three termination codons, while in bacteria there are two different codon-recognizing release factors, RF1 and RF2, which recognize UAA/UAG and UAA/UGA, respectively [14]. eRF1, categorized as a class 1 RF, recognizes the termination codon at the A site and promotes hydrolysis of peptidyl-tRNA at the P site of the ribosome. eRF1 also binds to a class 2 RF, eRF3, in eukaryotes. eRF3 is a GTP-binding protein, whose GTPase activity requires the presence of eRF1. Although eRF3 is not essential for the termination reaction in mammalian cells [15], it functions cooperatively with eRF1 to ensure efficient translation termination. eRF3 is also involved in NMD by directly interacting with the SURF (SMG1/UPF1/eRF1/eRF3) complex and UPF2 [16] (see below; Figure 1).

eRF1 can be divided into three domains: domain 1 (N-terminal), domain 2 (middle), and domain 3 (C-terminal). The crystal structure of eRF1, solved by Song et al., has shown that while the universal GGQ motif at an exposed tip of domain 2, which resembles the aminoacyl acceptor stem of tRNA, does not affect either codon recognition or ribosome–eRF3 interactions, it is critical for hydrolytic activity [17]. The model also suggests that the conserved groove on domain 1, which resembles the anticodon loop of tRNA, is involved in codon recognition. Domain 3, on the other hand, is both structurally and functionally equivalent to the T stem of the tRNA molecule.

eRF3 can be divided into at least two regions, the N-terminal extension region and the functionally essential C-terminal region [18]. The C-terminal region contains a highly conserved GTP-binding motif and plays a role in translation termination, while the N-terminal extension region is poorly conserved and not essential for the termination process. Nonetheless, the N-terminal region is indicated to have several physiological functions, such as association with poly(A) binding protein and switch of the functional mode of eRF3 by controlling its stability [19,20]. Interestingly, the crystal structure has revealed that the overall C-terminal region forms a structure similar to that of other translation factors, EF-Tu and eEF1A. The C-terminal region can be further divided into three domains: the GTPase domain (domain 1) and the β-barrel-structured domains (domains 2 and 3). Domain 3 contains a conserved GRFTLRD motif, which plays a crucial role in eRF1 binding [18].

In an effort to further decipher the interactions between eRF1 and eRF3 and how eRF1 decodes stop codons, several labs have solved the structures of eRF1/eRF3 and eRF1/eRF3/GTP ternary complexes [21,22,23]. Their structural analyses suggest that domain 2 of eRF1 contacts the GTPase domain of eRF3. They also show that upon eRF3 binding, eRF1 changes its conformation, acquiring a tRNA-like structure. Thus, the eRF1/eRF3/GTP complex mimics a tRNA/eEF1A/GTP ternary complex [24]. The former recognizes a nonsense codon, and the latter recognizes a sense codon during translation elongation. After the eRF1/eRF3/GTP complex enters the ribosome to recognize the nonsense codon at the A site, eRF3 dissociates in a GTP hydrolysis-dependent manner, allowing domain 2 of eRF1 to swing to the nonsense codon, and induces hydrolysis of the ester bond of the peptidyl-tRNA. Then, the ATPase ABCE1, which is a member of the ATP-binding cassette (ABC) family and is required for both translation initiation and ribosome recycling, binds to the ribosome–eRF1 complex and stimulates peptide release [23]. Subsequently, in the presence of ABCE1, eRF1 undergoes another conformational change, leading to post-termination complex dissociation. 

## 3. Nonsense-Mediated mRNA Decay Pathway

NMD is one of the most evolutionarily conserved and most extensively studied cellular quality control pathways. It has been shown that point mutations can create a PTC within a gene, and consequently the PTC-containing transcript is likely very susceptible to NMD. It is thus critical that NMD factors are able to distinguish target mRNA transcripts from non-target transcripts. 

Recent studies indicate that there are two different mechanisms of target selection, i.e., 3′ untranslated region (UTR) exon junction complex (EJC)-dependent NMD and 3′ UTR EJC-independent NMD [3,25,26,27]. Here, we will focus on the former, given that it is the major and the most efficient NMD pathway (Figure 1).

In 3′ UTR EJC-dependent NMD, the position and the distance of the PTC appear to be the crucial factors for target recognition and NMD activation. The Maquat group has proposed a “50–55 nucleotide rule,” which suggests that the NMD pathway is induced if the PTC is located more than 50–55 nucleotides upstream of the last exon–exon junction [28,29].

In addition, pre-mRNA splicing is a prerequisite for 3′ UTR EJC-dependent NMD [30]. Indeed, it was demonstrated that a PTC, when introduced into an intronless gene, failed to elicit NMD [31]. EJC consists of, among other components, four core proteins, including a DEAD-box helicase eIF4A3, two RNA-binding proteins Y14 and MAGOH (which form a heterodimer), and a nucleo-cytoplasmic protein, Metastatic Lymph Node 51 (MLN51) [32]. There are many other additional peripheral factors. Among them is a protein called RNPS1 (RNA-binding protein with serine-rich domain 1), the abundance of which correlates with NMD efficiency [33].

Perhaps the most important regulator of NMD is UPF1, which is an RNA-dependent helicase with ATPase and mRNA binding properties [34]. The ATPase and the helicase activities are stimulated by interactions with two other UPF proteins, UPF2 and UPF3B (in vertebrates, there are two UPF3 paralogs, UPF3A and UPF3B) [35,36]. Other key players of NMD are the SMG (Suppressor of Morphogenesis in Genitalia) proteins. SMG1 belongs to the phosphatidylinositol 3-kinase-related kinase (PIKKs) family and works as a serine/threonine kinase to phosphorylate UPF1, a critical step in NMD [37]. SMG1 also binds with SMG8 and SMG9. When the SMG1/SMG8/SMG9 complex forms, the kinase activity of SMG1 is inhibited [38,39]. Recently, the Cryo-EM structure of the SMG1/SMG8/SMG9 complex was determined and indicated that SMG1 kinase activity is inhibited by SMG8 C-terminal kinase inhibitory domain, while GTP hydrolysis by SMG9 would trigger a dramatic conformational change of the SMG8/SMG9 complex, promoting SMG1 kinase activity [40].

The 3ʹ UTR EJC-mediated NMD is generally initiated soon after a spliced mRNA is exported to the cytoplasm. For example, the PTC-containing β-globin mRNA has a half-life of less than 1 min, while the half-life of a normal β-globin mRNA is >12 h [41]. When the translating ribosome stalls at the PTC, the eRF1–eRF3 complex recruits both SMG1 and UPF1 to form the SURF complex. In the SURF complex, a conformational change in the SMG8/SMG9 heterodimer leads to restoration of SMG1 kinase activity, which allows SMG1 to phosphorylate UPF1 while interacting with the downstream 3ʹ UTR EJC. This SURF/EJC complex is called decay-inducing (DECID) complex [42], which directly recruits SMG6 or the SMG5/SMG7 heterodimer [43,44], leading to two different pathways. In the SMG6-mediated reaction, the endonuclease SMG6 cleaves the nonsense mRNA near the PTC [43]. In the alternative SMG5/SMG7-mediated pathway, upon directly interacting with POP2, SMG7 recruits the CCR4–NOT deadenylase complex, which removes the poly(A) tail and triggers deadenylation-dependent decapping and 5′-to-3′ decay [44]. The endonucleolytically or exonucleolytically cleaved mRNAs are then subject to several RNA degradation pathways, such as 5ʹ-to-3ʹ cleavage by exoribonuclease XRN1 and 3′-to-5′ cleavage by the exoribonuclease complex exosome [45,46]. Thus, PTC-containing mRNAs are rapidly degraded through the 3′ UTR EJC-mediated NMD pathway.

## 4. Suppression of Nonsense Mutations: Approaches and Challenges

It is reported that over 2400 different genetic diseases (corresponding to ~10% of all patients suffering from major genetic diseases) are caused by at least one nonsense mutation or a haplotype carrying it [47,48]. Suppression of such nonsense mutations is an attractive potential therapeutic approach (Figure 2). 

### 4.1. Nonsense Suppression by Small Molecules

Aminoglycoside antibiotics have long been suggested as a way to promote PTC recognition by near cognate tRNA, essentially competing with translation termination [49] and enabling the synthesis of a full-length protein [50,51]. Despite initial concerns with toxicity and side effects, these molecules have shown some success in clinical trials. Unfortunately, however, aminoglycosides are not equally efficient for all stop codons and perhaps work in a sequence context-dependent manner [52], thus limiting their applicability. The mechanism of action of aminoglycosides is not always entirely clear, although new methods, such as ribosomal profiling techniques that take a snapshot of all translating mRNAs, have been developed and can probably change this landscape and generate better lead molecules [53]. Additionally, these molecules are of great help as experimental tools in the preclinical development of new nonsense therapies [54]. For instance, Xue et al. were able to identify a new synthetic aminoglycoside (NB124) for the restoration of full-length CFTR (Cystic Fibrosis Transmembrane Conductance Regulator) protein in CF models whose CFTR gene contains nonsense mutations [55]. Using NB124, Xue et al. were able to restore CFTR protein function (7% of that of the wild-type protein) in human CF cells and in a CFTR knockout mouse model expressing a human CFTR–G542X transgene. This molecule was tested in healthy patients for safety and tolerability in phase I clinical trials by Eloxx Pharmaceuticals, under the designation ELX-02. The trials have shown some encouraging results, even if with some small side effects [56]. An upcoming phase 2 study with CF patients with at least one copy of the PTC-containing *CFTR* gene will further assess the efficacy and safety of this drug.

Another class of small molecules identified as nonsense suppressors are the oxadiazole derivatives, structurally unrelated to aminoglycosides. One example is Ataluren, which is one of the few clinically approved nonsense suppression drugs for Duchenne muscular dystrophy patients. Ataluren, initially called PTC124 {3-[5-(2-fluorophenyl)-[1,2,4]oxadiazol-3-yl]-benzoic acid; C_15_H_9_FN_2_O_3_}, was discovered by Welch et al. more than a decade ago [12]. Although the authors have shown that Ataluren has a dose–response nonsense suppression effect in a human cell line and that it is PTC-specific, the exact mechanism of action remains unclear. It is proposed that Ataluren targets the ribosome [13] and promotes the entry of a near-cognate tRNA into the A site of the ribosome to recognize the PTC, resulting in PTC read-through. Several in silico studies and reporter cell-based assays have shed additional light on the mechanism of action of Ataluren and structurally similar molecules with read-through activity [57,58,59]. Ataluren is also shown to have a similar effect in Usher syndrome patient-derived cells expressing a nonsense mutation in the *USH2A* gene [7]. Usher syndrome causes deaf-blindness in humans. Other oxadiazole derivatives have likewise been developed as potential nonsense suppression therapies and are reviewed elsewhere [60]. 

The search for new small molecules as potential nonsense suppression drugs continues, even outside the scope of aminoglycosides and oxadiazole derivatives. Recently, it was reported that clitocine and 2,6-diaminopurine, found in extracts from the mushroom *Lepista inversa*, have a significant nonsense suppression activity [61]. More specifically, 2,6-diaminopurine could allow UGA PTCs to be read as a tryptophan codon [62]. As with all small-molecule nonsense suppressors, it needs to be determined whether the amino acid incorporated at the PTC will render a full-length functional protein. 

The development of small molecules for nonsense suppression therapies can benefit from decades of R&D experience in the pharmaceutical industry. Small molecules are usually administered orally and have a good tissue distribution, achieved by systemic delivery. However, they can form non-specific molecular interactions with undesired targets, even beyond the nonsense suppression scope, leading to toxicity. There is therefore a need for new approaches that combine a simpler rational design with a higher degree of target specificity and potentially a longer half-life for an increased duration of the effect, allowing fewer or even single treatments. 

### 4.2. Nonsense Suppression by Nucleic Acid-Based Approaches

The emerging nucleic acid-based technologies are the alternatives to small molecules for nonsense suppression. There are a number of mechanistically different approaches that have been reported, and here, we review some of them, focusing on antisense oligonucleotides, suppressor tRNA, Adenosine Deaminase acting on RNA (ADAR)-catalyzed RNA editing, as well as box H/ACA guide RNA-directed pseudouridylation and CRISPR technology.

#### 4.2.1. Antisense Oligonucleotides

Antisense oligos with exon-skipping ability were suggested two decades ago as a means to splice out PTC-harboring exons in frame [63,64,65]. However, this approach has a potential disadvantage, in that the alternatively spliced (exon-skipping) transcript may encode a non-functional protein, which needs careful assessment on a case-by-case scenario [66,67]. That said, it could be a valuable alternative, especially for diseases where gene replacement therapies prove very difficult, possibly because of the large size of the gene (difficult to be delivered to patients).

Several labs have used antisense oligos, in the form of gapmers, as a way of downregulating specific protein components of the NMD pathway, such as Upf3b [68] and SMG1 [69], to suppress NMD. Likewise, it has also been reported that gapmer-mediated downregulation can be used to target the release factors eRF1 and eRF3A, thus suppressing translation termination at the PTC [70]. An alternative strategy has also been published, where antisense oligos are used to sterically block the formation of the EJC located next to the PTC [71]. 

#### 4.2.2. Suppressor tRNA

Another nucleic acid-based strategy for nonsense suppression relies on tRNA suppressors that would act by incorporating a cognate amino acid at the PTC position [5]. Recently, Lueck et al. [72] devised a high-throughput strategy to engineer artificial tRNAs, which would still be recognized by the endogenous aminoacyl tRNA synthetases and elongation factor, to promote the recognition of three different stop codons by cognate suppressor tRNAs. This strategy is also being developed at the preclinical level by ReCode Therapeutics, in that the researchers have designed a modified tRNA (RCT101) to incorporate an amino acid at known CFTR PTC positions, with the aim of restoring a functional CFTR protein (or a functional chloride ion channel) [73]. Despite the potential risks of this approach, namely, potential off-target effects created by an amino acid mis-incorporation at the normal stop codons, it is a good addition to the toolbox of ways to recode the human transcriptome.

#### 4.2.3. ADAR-Catalyzed Editing Directed by Guide RNA

Ever since Woolf et al. [74] proposed the use of antisense oligonucleotides to recode a PTC in a dystrophin mRNA construct in ADAR-containing extracts prepared from human cell nuclei and in Xenopus embryos, this field has attracted immense attention. This strategy relies on the assumption that antisense oligonucleotides are able to direct ADAR to edit an adenosine of the PTC codon and convert it into an inosine, which is read as guanosine by the translation machinery. This scenario would be ideal in PTC-related diseases in which the PTC results from a G-to-A mutation (e.g., UGG changes to UAG). By site-specifically changing A to I, one would reverse the PTC back to the wild type.

This concept of site-specific A-to-I editing has been further advanced by Montiel-Gonzalez et al., as shown in the functional correction of CFTR nonsense mutations [75] in Xenopus oocyte membranes. These authors have initially developed a system where an engineered ADAR editing machinery is co-delivered with a guide RNA (gRNA) targeting the G-to-A mutation via a λ-phage N protein-boxB RNA. In parallel, others have proposed simpler and more elegant A-to-I editing approaches. For instance, by attaching an hairpin structure, resembling a natural ADAR substrate, to the antisense guide RNA, Wettengel et al. were able to recruit endogenous or exogenously expressed human ADAR2, a member of the ADAR family, to recode a PTC into the sense codon (the tryptophan codon) [76]. This strategy can be implemented with gRNAs being delivered as chemically modified oligos [77] or encoded in AAVs (Adeno-associated viruses) under the control of the U6 promoter [78].

It is well established that endogenous RNA editing occurs mostly in the nucleus via the ADAR1 p110 isoform and ADAR2. The other form of ADAR1 is ADAR1 p150, which is interferon-inducible and found both in the nucleus and in the cytoplasm [79]. Some of the proposed ADAR-mediated PTC corrections have been shown in settings where ADAR is transiently expressed. This is often achieved by employing a hyper-active mutant version of ADAR2, such as E488Q [80]. To achieve efficient editing in the cytoplasm (with the help of antisense oligonucleotides), the hyper-active mutant ADAR is often expressed without being targeted to the nucleus (lacking a nuclear localization signal (NLS). However, these mutant ADAR versions could lead to significant off-target effects. To mitigate this risk, Monteleone et al. showed that they were able to reduce the off-target effects by mutating ADAR2 and altering the guide RNA [81]. Vallecillo-Viejo et al. also showed that the off-target effects could be reduced when artificial versions of ADAR were targeted to the nucleus (through attaching several NLS to the artificial ADAR) [82]. Interestingly, some studies also demonstrated the recoding of UAG codons present in the 3′-UTR of several genes in human cells, and it appears that this is easier to achieve as compared to a true PTC [77].

Because the NMD pathway dramatically reduces the mRNA substrate level, without inhibiting the degradation of PTC-containing mRNA, the supply of substrate that can be recoded could be limited. Although gRNA-directed ADAR editing can in theory suppress NMD as well, experimental verification remains necessary. Recently, using the editing approach, Qu et al. have restored the full-length iduronidase (IDUA) protein in human skin fibroblasts harboring a common nonsense mutation in the *IDUA* gene (W402X) [83]. Editing appears to be more efficient in pre-mRNA than in mature transcripts, underscoring the need for approaches that inhibit the NMD pathway to increase the amount of mRNA and, ultimately, of functional proteins (Hsiao et al. observed that >95% of A-to-I RNA editing events occurred in chromatin-associated RNA prior to polyadenylation) [84].

Finally, it is worth noting that this editing strategy is highly dependent on the sequence and secondary structure contexts of the target site. For instance, there is a preference for certain 5′ and 3′ flanking nucleotides depending on the ADAR type [85].

#### 4.2.4. RNA Pseudouridylation Directed by Box H/ACA Guide RNA

Karijolich and Yu have shown that the targeted conversion of uridine in the first position of a PTC into pseudouridine, a C-5 glycoside isomer of uridine (but chemically and structurally distinct), results in nonsense suppression in vitro and in yeast cells [86].

Targeted pseudouridylation can be catalyzed by an RNA–protein complex known as box H/ACA ribonucleoprotein (RNP) (consisting of one RNA and four core proteins, i.e., Cbf5, Nop10, NHp2, and Gar1). Specifically, the RNA component (box H/ACA RNA) serves as a guide that base-pairs with the substrate RNA, specifying the target uridine, which is subsequently modified by Cbf5/NAP57, the catalytic component of the complex. The guide RNA of each box H/ACA RNP folds into a hairpin–hinge–hairpin–tail secondary structure, with one internal loop in each hairpin. The internal loop sequence, also referred to as pseudouridylation pocket, forms base-pairing interactions with the substrate sequence, specifying the target uridine to be recognized and modified by Cbf5/NAP57. In principle, one can change only the guide sequence of a naturally occurring box H/ACA RNA to target a uridine at any position in an RNA. Upon expression in the cell, the guide RNA forms a box H/ACA RNP complex with the endogenous box H/ACA RNP proteins. Indeed, Karijolich and Yu have shown that they were able to design an artificial box H/ACA guide RNA derived from SNR81, a naturally occurring yeast H/ACA RNA, to direct pseudouridylation of a PTC (for review, see [87]). 

It seems that this read-through mechanism of action works by promoting unusual interactions between the codon–anticodon pair, which outcompete the release factors in binding to the PTC [88]. Furthermore, Karijolich and Yu were able to determine the identity of the amino acids inserted in the recoded PTC via immunoprecipitation and mass spectrometry: threonine or serine was incorporated at the ΨAA and ΨAG codons, and phenylalanine or tyrosine at the ΨGA codons. These results support the hypothesis that pseudouridylation affects the coding rules in a specific manner. 

The guide–substrate base-pairing requirements have been recently analyzed, and it appears that the pseudouridylation pockets are flexible enough to be engineered to recognize virtually any target RNA, while specificity can be achieved with a minimum number of eight base pairs between the guide and the substrate [89,90]. This novel mechanism of action has a significant therapeutic potential, since the artificial box H/ACA guide RNAs can be designed to recode, with a high degree of sequence specificity, virtually any therapeutically relevant PTC, with no sequence–context dependency and likely little or no off-target effects.

Another advantage of this approach is the fact that an artificial guide RNA, upon expression in the cell, complexes with the endogenous proteins to form a functional box H/ACA RNP. Thus, this approach requires the delivery and expression of only one small guide RNA; no co-expression of other gene(s) is necessary. In a therapeutic setting, nonsense-suppressing designer box H/ACA snoRNAs could be administered as chemically modified oligonucleotides (which have proven to be functional in vitro) for a short- to mid-term effect. Alternatively, the guide RNA can be inserted into viral vectors, such as adeno-associated viruses, for a more prolonged pharmacological effect in the target tissue. 

#### 4.2.5. CRISPR Technology

The advent of genome editing technologies [91], particularly CRISPR gene editing, has opened an entirely new field with numerous opportunities for potential nonsense suppression therapies. Despite the heavily discussed challenges and ethical concerns around these technologies, the first human patient was recently dosed with a CRISPR-based gene therapy for the treatment of Leber’s Congenital Amaurosis Type 10 (LCA10) in a clinical trial sponsored by Editas Medicine and Allergan. The drug, consisting of a nuclease and two guide RNAs, was delivered with the help of an adeno-associated virus to correct a splicing mutation in the *CEP290* gene, which causes the disease. This first treatment is a scientific and technological landmark, as it shows that a groundbreaking and a quite complex treatment for the repair of genetic disorders could reach patients in less than a decade.

These technologies also hold great promise for the correction of nonsense mutations, whether through an active Cas9-derived nuclease that will directly cut and paste the genome or through the use of base editors, i.e., deactivated versions of the nuclease coupled to an adenosine deaminase. Base editors can either correct the genome without introducing double-strand breaks or specifically target a mutated mRNA. Lee et al. have recently presented an example of a DNA-targeting CRISPR-based tool with a version of Cas9, named CRISPR-PASS, for the correction of therapeutically relevant nonsense mutations [92]. In this study, they achieved read-through in skin-disorder (xeroderma pigmentosum complementation group C, XPC) patient-derived fibroblasts expressing the *XPC* gene with a nonsense mutation (1840C > T, Arg579X). Base editing efficiencies were 10%, and additional functional restoration of the protein was observed for up to one month after treatment. In another study, Cox et al. provided an example of RNA-targeting repair of PTCs. They fused the catalytic domain of ADAR2 with a deactivated Cas13b, a nuclease that can target RNA instead of DNA, to achieve editing in nonsense mutations of two genetic disorders (X-linked nephrogenic diabetes insipidus and Fanconi anemia) in substrates exogenously delivered via transfection [93]. Although there is still a long way to go, these results are promising. 

## 5. Concluding Remarks

Over the years, a number of approaches have been developed to suppress nonsense mutations. These approaches can be roughly grouped into two categories: small-molecule-based approaches and nucleic acids-based approaches. Both have advantages and limitations. For instance, small-molecule-based approaches are simple and easy when it comes to cellular delivery. However, given that they presumably target the ribosomes (the mechanisms of action are often unclear), small molecules are probably not PTC target-specific. In contrast, despite the fact that nucleic acids are macromolecules (relatively large) and are therefore not always readily taken up by the cells, nucleic acids-based approaches are developed based on known mechanisms of action, with clear rationale and objectives. Most of the nucleic acids-based approaches specifically target PTC-containing mRNA transcripts (and the PTC) and therefore are relatively sequence-specific.

It should be noted that all the different approaches discussed above are capable of suppressing NMD, translation termination, or both. Importantly, many of them have already been shown to be effective in nonsense suppression at least at the cellular level (some of them have even passed preclinical and clinical trials). Thus, they have recently attracted a lot of attention. It is expected that with various approaches being developed in parallel, some will succeed and become effective therapies. The race is on towards a cure for nonsense-associated diseases.
